# Multiple extremity necrosis in fatal calciphylaxis: Case report

**DOI:** 10.1590/2175-8239-JBN-2020-0025

**Published:** 2020-07-08

**Authors:** Diego Ennes Gonzalez, Renato Demarchi Foresto, Ana Luiza Santos Maldonado, Wallace Stwart Carvalho Padilha, Fernanda Badiani Roberto, Maria Eduarda Vilanova da Costa Pereira, Marcelino de Souza Durão, Aluizio Barbosa Carvalho

**Affiliations:** 1 Universidade Federal de São Paulo (UNIFESP), Escola Paulista de Medicina (EPM), Hospital do Rim, São Paulo, SP, Brasil.; 2 Universidade Federal de São Paulo (UNIFESP), Escola Paulista de Medicina (EPM), Departamento de Medicina, São Paulo, SP, Brasil.

**Keywords:** Vascular Calcification, Renal Insufficiency, Chronic, Calciphylaxis, Monckeberg Medial Calcific Sclerosis, Calcificação Vascular, Insuficiência Renal Crônica, Calciofilaxia, Esclerose Calcificante da Média de Monckeberg

## Abstract

**Introduction:**

The clinical impact of vascular calcification is well established in the context of cardiovascular morbidity and mortality, but other clinical syndromes, such as calciphylaxis, although less frequent, have a significant impact on chronic kidney disease.

**Methods:**

Case report of a 27-year-old woman, who had complained of bilateral pain in her toes for 3 days, with the presence of small necrotic areas in the referred sites. She had a history of type 1 diabetes (25 years ago), with chronic kidney disease, on peritoneal dialysis, in addition to rheumatoid arthritis. She was admitted to the hospital, which preceded the current condition, due to exacerbation of rheumatoid arthritis, evolving with intracardiac thrombus due to venous catheter complications, when she started using warfarin. Ischemia progressed to her feet, causing the need for bilateral amputations. Her chirodactyls were also affected. Thrombophilia, vasculitis, endocarditis or other embolic sources were investigated and discarded. Her pathology report evidenced skin necrosis and superficial soft parts with recent arterial thrombosis, and Monckeberg's medial calcification. We started treatment with bisphosphonate and sodium thiosulfate, conversion to hemodialysis and replacement of warfarin with unfractionated heparin. Despite all the therapy, the patient died after four months of evolution.

**Discussion:**

Calciphylaxis is a rare microvasculature calcification syndrome that results in severe ischemic injuries. It has pathogenesis related to the mineral and bone disorder of chronic kidney disease combined with the imbalance between promoters and inhibitors of vascular calcification, with particular importance to vitamin K antagonism.

**Conclusion:**

The preventive strategy is fundamental, since the therapy is complex with poorly validated effectiveness.

## RESUMO

**Introdução:**

O impacto clínico da calcificação vascular está bem estabelecido no âmbito de morbimortalidade cardiovascular, mas outras síndromes clínicas, como a calcifilaxia, apesar de menos frequente, têm significante impacto na doença renal crônica.

**Métodos:**

Relato de caso de mulher, 27 anos, com queixa de dor em pododáctilos bilateralmente havia 3 dias, com presença de pequenas áreas necróticas nos locais referidos. Antecedente pessoal de diabetes tipo 1 (há 25 anos), com doença renal crônica, em diálise peritoneal, além de artrite reumatoide. Teve internação hospitalar, que antecedeu o quadro atual, devido à exacerbação da artrite reumatoide, evoluindo com trombo intracardíaco por complicação de cateter venoso, quando iniciou uso de varfarina. A isquemia progrediu para pés com necessidade de amputações bilaterais. Quirodáctilos também foram acometidos. Trombofilias, vasculites, endocardite ou outras fontes emboligênicas foram pesquisadas e descartadas. Anatomopatológico evidenciou: necrose de pele e partes moles superficiais com trombose arterial recente e calcificação medial de Monckeberg. Tratamento foi instituído com bisfosfonato e tiossulfato de sódio, conversão para hemodiálise e substituição de varfarina por heparina não fracionada. Apesar de toda a terapia, a paciente foi a óbito após quatro meses de evolução.

**Discussão:**

A calcifilaxia é uma rara síndrome de calcificação da microvasculatura que resulta em graves lesões isquêmicas. Tem patogênese relacionada ao distúrbio mineral e ósseo da doença renal crônica combinado com o desbalanço entre promotores e inibidores de calcificação vascular, com particular importância ao antagonismo da vitamina K.

**Conclusão:**

A estratégia preventiva é fundamental, uma vez que a terapia é complexa e de eficácia pouco validada.

## INTRODUCTION

Vascular calcification (VC) is a degenerative process resulting from deposits of phosphate and calcium salts on the artery wall, with consequent loss of its elasticity. While calcification of the arterial intima layer is associated with the atherosclerotic inflammatory process, located mainly in the aorta, coronary arteries and other large vessels, calcification of the middle layer, known as Monckeberg calcifying sclerosis, has precipitating factors, such as age, diabetes and chronic kidney disease.^1^ VC is not only a passive process of mineral deposition, but mainly an active process of altering protein regulation that modifies smooth muscle vascular cells in simile osteoblasts.^2^ Such differentiation is determined by the balance between mediating factors promoting and inhibiting calcification.^3^

The clinical impact of VC is well established in the context of cardiovascular morbidity and mortality, present since the early stages of chronic kidney disease (CKD).^4^^,^ 5 Although less frequent, calciphylaxis represents a spectrum of this condition of undoubted importance, of diagnostic-therapeutic management, challenging, significant lethality and profound impairment of the patient’s quality of life in renal replacement therapy.^6^

Next, we describe a case of a patient with atypical and severe evolution of calciphylaxis in concomitance with Monckeberg sclerosis, followed by a literature review.

## CASE REPORT

A 27-year-old female patient entered the emergency department complaining of pain in her toes for 3 days, especially in the first right toe, associated with skin browning there. He progressed rapidly to pain on the contralateral side of the second toe, which motivated the demand for care.

Among the patient’s personal history, the following stood out: type I diabetes mellitus for 25 years, rheumatoid arthritis for 15 years and chronic kidney disease in renal replacement therapy 9 months after presentation - initially hemodialysis, remaining for four months, being converted to peritoneal dialysis due to vascular access difficulties. In the same year, she was hospitalized (three months before the current admission) due to exacerbation of rheumatoid arthritis and mitral valve endocarditis, triggered by a central venous access, with satisfactory clinical, microbiological and echocardiographic response to antimicrobial therapy. At that time, the presence of a right atrial thrombus was also diagnosed and full oral anticoagulation was started with a vitamin K antagonist (warfarin). In addition, she used the following medications: insulin, hydralazine, clonidine, leflunomide, prednisone, erythropoietin, atorvastatin and calcium carbonate. Life habits and family history were irrelevant.

**Figure 1:**
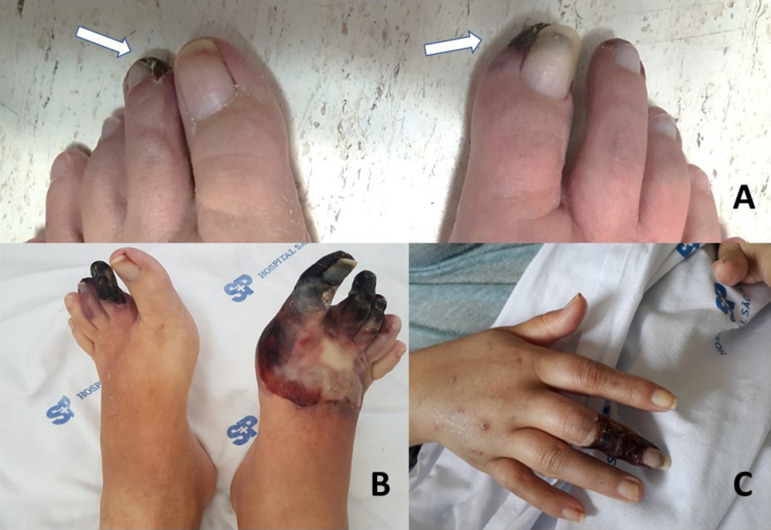
Initial aspect (A - white arrows) and evolution of the lesion in 1 month (B) and in 3 months (C).

Upon physical examination, there were no changes in vital parameters or other cardiopulmonary peculiarities, and her body mass index was 26 kg/m². The posterior popliteal, pedicle and tibial pulses were impalpable bilaterally, with peripheral perfusion present, but slowed. Foot exam showed distal necrotic lesions in the second left toe and first right toe (Figure 1-A).

Laboratory tests showed: hemoglobin 7.5 g/dL, leukocytes 22,100 (RV: 4,000-10,000), platelets 463,000 (RV: 150,000-450,000), C-reactive protein (CRP) 21.39 mg/dL (RV: < 5.00), urea 156 mg/dL, ionic calcium 1.10 mmol/L (RV: 1.10-1.40), phosphorus 7.5 mg/dL (RV: 2.5-4.5), international normalized ratio (INR) 2.08 (RV: 1.25). Initially suspected of arterial embolization, the probable cardiogenic source was investigated by a transesophageal echocardiogram, which showed a thrombus attached to the right atrium wall (measuring 18 x 30 mm - with characteristics similar to the examination performed three months earlier), with no signs of valve pedicles. Blood cultures were negative and ocular fundoscopy showed chronic proliferative diabetic retinopathy. Her chest radiograph was normal.

Once the possibility of endocarditis from other embolic sources was ruled out, arterial angio-CT of the lower limbs showed irregularities in the distal circulation, with diffuse atheromatous, without evidence suggestive of acute arterial thromboembolism. Without diagnostic conclusion and with the progression of the lesions (Figure 1-B), in a previous context of rheumatological disease, we ran other tests: antinucleus factor (ANA), rheumatoid factor, cryoglobulins, antibodies against the neutrophil cytoplasm (ANCA), lupus anticoagulant, anticardiolipins, all of which were non-reactive. Complement C3 and C4 fractions were within normal limits.

**Figure 2:**
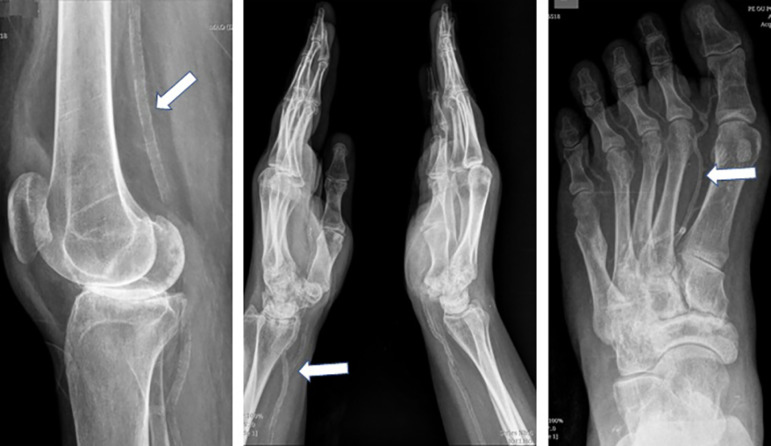
Simple X-Rays showing extensive calcification in the arteries (white arrows).

Her evolution was severe and fast. After undergoing right transfemoral amputation, 90 days after the initial condition, she needed contralateral transfemoral amputation as well. The pathology exam showed recent arterial thrombosis, Monckeberg’s medial calcification, skin necrosis and superficial soft tissues, in addition to morphological findings suggestive of calciphylaxis. This context prompted a retrospective investigation of the patient for a better understanding of the important vascular involvement. Then, we assessed radiographs of the limbs, taken at the beginning of the same year, in a rheumatology clinic, in which there was extensive calcification in the arterial territory of the upper and lower limbs (Figure 2). In parallel, there was an evolution of the laboratory parameters of the mineral and bone disorder, since the beginning of follow-up by nephrology, when still under conservative treatment (Table 1).

**Table 1:** Laboratorial profile of the bone-mineral disorder. Cai = ionic calcium, P = Phosphorus, FA = alkaline phosphatase, PTH = parathyroid hormone. "0" means the month in which the lesions started. The negative values mean the months that preceded the clinical picture described; the positive values mean the months afterwards.

Month	-11	-10	-9	-8	-7	-6	-5	-4	-3	-2	-1	0	1	2	3
Cai (mmol/l)	1,21	1,23	1,15	1,33		1,12	1,09	0,88	0,87	0,96	0,84	1,05	1,25	1,3	1,4
P (mg/dL)		5,4	4,7	2,9	4,3	4,8	6,7	8,5	8,8	12,1	11,6	8,1	3,8	6,2	7,2
FA (U/L)		173				154				122		154	83		
PTH (pg/mL)		256			449	649				491	800		341		

Regarding the instituted therapy, there was a conversion from peritoneal dialysis to intermittent hemodialysis, intravenous pamidronate infusion, sodium thiosulfate, warfarin suspension (being replaced by unfractionated heparin in continuous infusion), in addition to optimized analgesia with opioids and local anesthetic block. Despite all the measures adopted, in prolonged hospitalization, the patient complicated with various nosocomial infections, evolving to death.

## DISCUSSION

Monckeberg’s sclerosis was initially described as a medial arterial calcification that affected elderly patients. However, at the time, histopathological findings were unknown, nor associated with the renal function of the patients studied. Subsequently, there was an evolutionary and advanced stage of a calcifying atherosclerotic process, with minimal inflammatory process, and in the literature, the term has been constantly linked to the clinical condition of calciphylaxis. Therefore, Monckeberg’s medial sclerosis is a manifestation of calciphylaxis superimposed on systemic atherosclerosis; a continuum of extra-skeletal osteogenesis, which predominant clinical features are: skin necrosis, soft tissue calcification and severe peripheral ischemia, requiring amputation.^7^

Calciphylaxis is a rare vascular calcification syndrome characterized by occlusion of the subcutaneous microvasculature, resulting in painful ischemic lesions of a heterogeneous clinical spectrum. Typically present in stage 5D CKD, its incidence reaches about 35 cases for every 10,000 patients on dialysis, yielding worrying prognosis, with mortality rates greater than 50% .^6^ There are several risk factors, linked or not to CKD, with emphasis on: female gender, obesity, diabetes, hypoalbuminemia, use of warfarin and bone mineral disorder - especially adynamic bone disease and hyperphosphatemia.^8^^,^ 9 High levels of parathyroid hormone (PTH) are not associated with a higher incidence of calciphylaxis.^10^^,^ 11 The role of vitamin K is particularly important in this condition. The matrix Gla protein (MGP), in its carboxylated form, is a potent inhibitor of vascular calcification, a vitamin-K-dependent process. Concomitantly, carboxylated MGP inhibits the morphogenetic bone procalcifying proteins (BMP). Therefore, by inhibiting the pharmacological action of warfarin, MGP triggers an important role in the development of calciphylaxis.^12^

Ischemic lesions of proximal (abdomen, thighs) or distal (legs, fingers), characterize the clinical picture topography, initially with purpuric and erythematous characteristics; with time, they become ulcerated and necrotic. When fully present, one can confirm the diagnosis using the following criteria: (a) CKD, on dialysis, or glomerular filtration rate less than 15 mL/min/1.73 m²; (b) more than two painful ulcerated lesions, with purpura; (c) located on the trunk, extremities or penis.^13^ In the absence of one of the clinical criteria, one needs a histopathological analysis with Von Kossa staining, specific for tissue calcium deposits.^14^

The treatment of calciphylaxis is multidisciplinary and multidirectional, with the objective of fighting VC and thrombosis, injury management and analgesia. With regards to calcification, sodium thiosulfate, a substance with calcium chelating properties, antioxidant and vasodilatory effects in observational studies, has proven to be effective in resolving lesions, but with the proviso that there is a need for clinical trials, including one of them in progress (NCT03150420).^15^ There are also reports of improvements in lesions with the use of bisphosphonates (pamidronate), in addition to optimization of dialysis therapy. In addition to this, there is conversion to hemodialysis, if the patient is on a peritoneal dialysis program.^16^ The management of wounds with debridement of devitalized tissues, as well as analgesia, with optimization of opioids and adjuvants, is essential, since injuries can take on serious and disabling proportions.

The validation of therapies, when analyzed systematically, fails to show a positive impact on mortality, which urges the need for randomized trials.^17^ In addition to the therapeutic approach, it is essential to emphasize the need for a preventive approach to VC in patients with CKD, which goes beyond bone mineral disorder control. An example of this, vitamin K supplementation has shown positive results when studied in the context of coronary calcification,^18^ and its effectiveness in calciphylaxis is being tested prospectively (NCT02278692). In parallel, the role of magnesium as an “anti-calcifying” substance has been studied, with promising results, without significant adverse effects.^19^

This case illustrated, although rare, a severe and quickly compromising disease in a young patient, with a strong impact on quality of life and a fatal outcome. It alludes to an important problem in CKD, in which the multifaceted preventive and therapeutic management is, without a doubt, the alternative that aims to improve survival and quality of life of the population in question.
